# Eosinophilic granulomatosis with polyangiitis after treatment with dupilumab

**DOI:** 10.1016/j.jacig.2022.03.006

**Published:** 2022-04-30

**Authors:** Kai Yamazaki, Takafumi Nomizo, Kazuhito Hatanaka, Naoki Hayama, Tsuyoshi Oguma, Koichiro Asano

**Affiliations:** a Division of Pulmonary Medicine, Department of Medicine, Tokai University School of Medicine, Kanagawa, Japan; c Department of Pathology, Tokai University School of Medicine, Kanagawa, Japan; b Department of Medicine, Shonan-Atsugi Hospital, Kanagawa, Japan

**Keywords:** Dupilumab, eosinophilic granulomatosis with polyangiitis, severe asthma

## Abstract

Eosinophilic granulomatosis with polyangiitis (EGPA) in association with the use of asthma medications has been reported. We report the first Asian case of EGPA developed after dupilumab administration in a 77-year-old Japanese woman and discuss the association between dupilumab and EGPA.

Eosinophilic granulomatosis with polyangiitis (EGPA) is a type of systemic vasculitis that develops following prodromes such as asthma, chronic rhinosinusitis with nasal polyps (CRSwNP), and hypereosinophilia. More than half of the cases are antineutrophil cytoplasmic antibody (ANCA)-negative, and these cases may have genetic backgrounds, clinical characteristics, and therapeutic responses that are distinct from those of ANCA-positive cases.^1^ EGPA, adult-onset asthma, and CRSwNP are considered adult-onset eosinophilic airway diseases sharing common genetic predispositions.^2^

Vasculitic symptoms of EGPA in patients with severe asthma can emerge when oral corticosteroids are tapered following improvements in asthmatic symptoms thanks to other asthma medications, such as leukotriene receptor antagonists or omalizumab (an anti-IgE antibody).^3^ In contrast, mepolizumab, an anti–IL-5 antibody, promotes EGPA remission by suppressing the differentiation and activation of eosinophils. Whether dupilumab, an anti–IL-4 receptor-alpha antibody, causes the development of overt EGPA from latent diseases as omalizumab does or suppresses its emergence, similar to mepolizumab, is still unclear. We present a case of EGPA that developed 6 months after dupilumab treatment for severe asthma. The patient provided informed consent for this case report.

A 77-year-old Japanese woman with a history of asthma, chronic rhinosinusitis (CRS), and eosinophilic pneumonia was admitted to the hospital with fever, polyarthralgia, and skin rash. She was initially diagnosed with asthma with coughing, wheezing, peripheral blood eosinophilia (869 cells/μL), and decreased ratio of FEV_1_ to forced vital capacity (54%) 3 years prior. Combination therapy of inhaled corticosteroids and long-acting β_2_-agonist was started, and the patient's ratio of FEV_1_ to forced vital capacity returned to the normal level (80%) in 2 weeks. Nine months later, the patient presented with worsened cough accompanied by marked peripheral blood eosinophilia (5.967 cells/μL), increased serum IgE level (918 IU/mL), and ground-glass opacities in the bilateral upper lobes of the lungs on chest computed tomography. Oral prednisolone (PSL) at a dose of 0.5 mg/kg per day was started on the basis of clinical diagnosis of chronic eosinophilic pneumonia, after which the patient's cough and ground-glass opacities disappeared. Six months before admission, when asthma exacerbations became frequent despite the use of oral PSL (5 mg per day) and radiographic examination demonstrated CRS, dupilumab (600 mg as the loading dose followed by 300 mg every 2 weeks) was started. Asthma control improved, and the dose of PSL was slowly reduced to 2 mg per day. The patient's serum IgE level decreased to 198 IU/mL, although mild peripheral blood eosinophilia (695 cells/μL) persisted.

Fever (38°C), night sweating, arthralgia in the neck and the bilateral hip and knee joints, and weakness in the lower limbs appeared 1 month later. Erythematous plaques approximately 1 cm in diameter were observed in the patient's abdomen and upper limbs (Fig 1, *A* and *B*). Her peripheral blood eosinophil count (835 cells/μL), platelet count (54.6 × 10^4^/μL), and serum level of C-reactive protein (27.9 mg/dL) were significantly increased, whereas her myeloperoxidase- and proteinase 3–ANCA test results were negative. Head and body computed tomography showed bilateral ethmoid sinusitis, (Fig 1, *C*) but no pulmonary involvement. Positron emission tomography showed ^18^F-fluorodeoxyglucose accumulation in the patient's right shoulder and left hip joint (Fig 1, *D* and *E*). Magnetic resonance imaging showed mild edematous changes between the deep muscles of the lower right thigh (Fig 1, *F*). Pathologic examination of the skin rash revealed necrotizing phlebitis with modest eosinophil infiltration around the veins but no granuloma (Fig 2, *A* and *B*).

**Fig 1:**
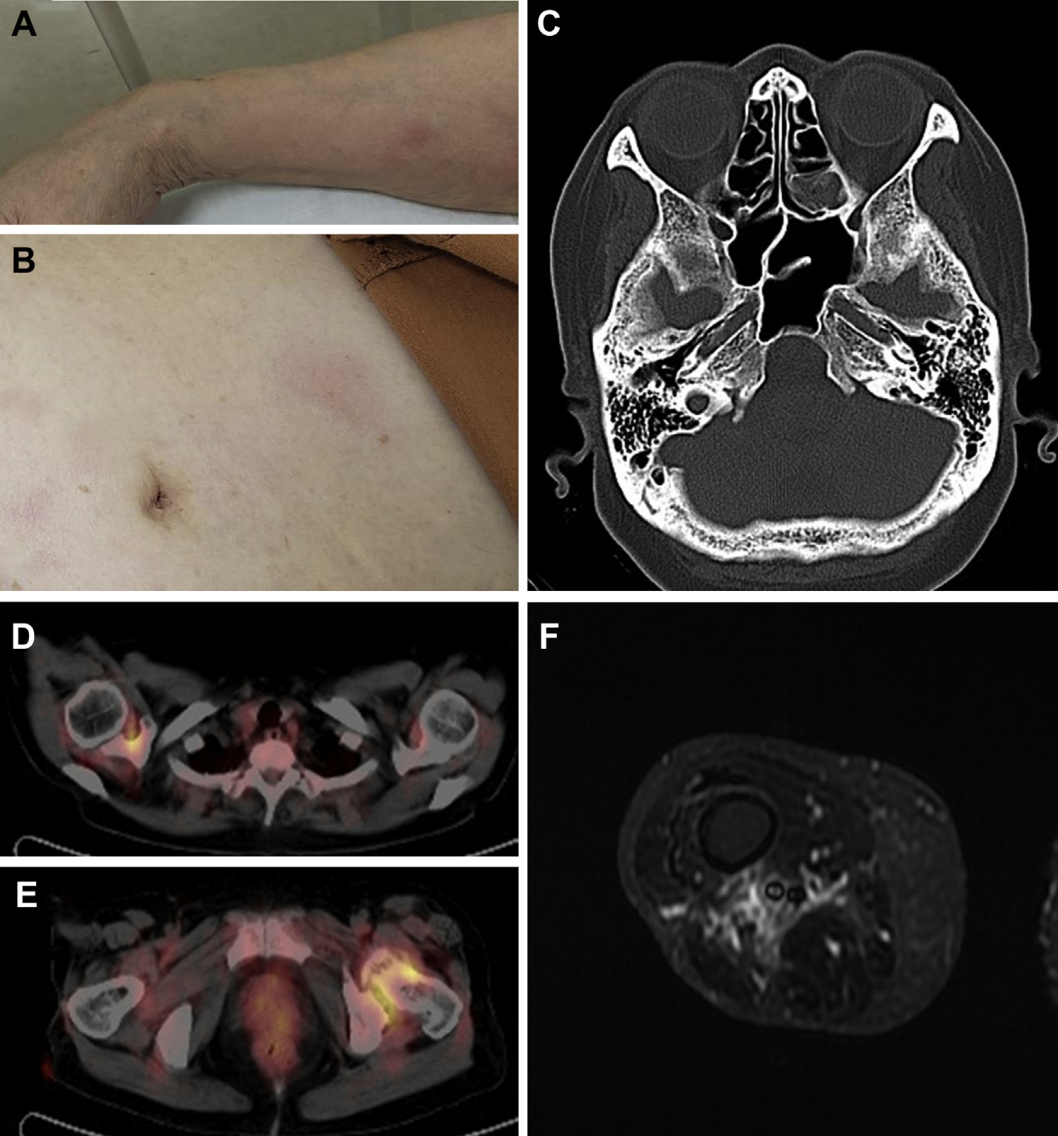
Skin rash and radiographic findings at time of admission. **A** and **B**, Erythematous plaques are visible on the upper limb (**A**) and abdomen (**B**). **C,** Sinus computed tomography image showing bilateral ethmoid sinusitis. **D** and **E,** Positron emission tomography showing ^18^F-fluorodeoxyglucose accumulation at the right shoulder (**D**) and left hip joint (**E**). **F,** Magnetic resonance imaging of the lower right thigh showing mild edematous changes between the deep muscles.

**Fig 2:**
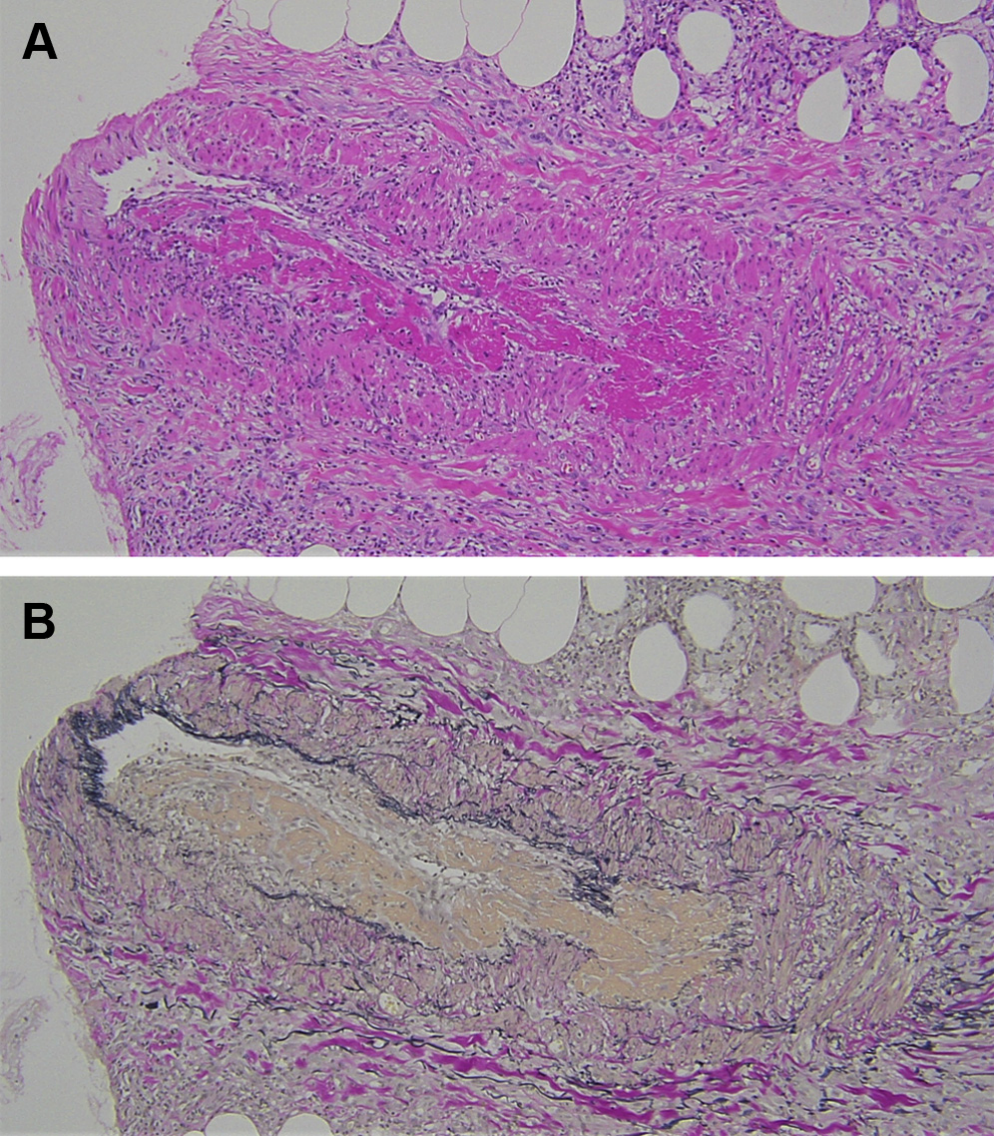
Pathologic examination of the skin rash on the upper extremity. Necrotizing phlebitis with a slight infiltration of eosinophils can be observed around the veins and in the adipose tissue. Granuloma is not present. **A,** Hematoxylin-eosin staining (original magnification, ×100). **B,** Elastica van Gieson staining (original magnification, ×100)

In this case, asthma, ethmoid sinusitis, eosinophilic pneumonia, and peripheral blood eosinophilia, followed by development of fever, polyarthritis, fasciitis, skin rash, and pathologic vasculitis with eosinophilic infiltration, fulfilled the 3 Lanham diagnostic criteria of EGPA^4^ and satisfied 5 of the 6 classification criteria of the American College of Rheumatology^5^: asthma, an eosinophil count greater than 10%, pulmonary infiltrates, sinus abnormalities, and extravascular eosinophils. Although the patient lacked multiple mononeuritis, which occurs in 90% of patients with EGPA, the patient’s symptoms and clinical course were consistent with those of an EGPA diagnosis. Dupilumab was discontinued, and the dose of oral prednisolone was increased to 1 mg/kg per day. The patient was discharged on day 34. Her PSL dose was gradually reduced to 6 mg per day, and her symptoms did not recur over the course of 10 months.

Dupilumab itself may have triggered the development of EGPA. Activated eosinophils play an important role in the pathogenesis of EGPA by causing tissue damage by granular proteins, reactive oxygen species, or extracellular trap cell death (ETosis).^6^ Dupilumab inhibits eosinophil infiltration and activation in airway tissues by blocking the expression of eosinophilic chemokines and adhesion molecules, but it does not suppress activated eosinophils in the peripheral blood, which may damage the vascular walls. A paradoxic increase in the peripheral blood eosinophil counts, which peaked at 12 weeks after dupilumab treatment,^7^ corresponds with the timing of EGPA development in our case (at 6 months).

Another possibility is that latent EGPA emerges during tapering of systemic corticosteroids or anti–IL-5 treatment with the introduction of dupilumab, as in cases of omalizumab-associated EGPA. Notably, all cases of dupilumab-associated EGPA have developed in patients with either asthma or CRSwNP. Eger et al reported the case of a 63-year-old woman with asthma who developed EGPA 8 weeks after switching from an anti–IL-5 receptor antibody to dupilumab.^8^ Another possible case of dupilumab-associated EGPA occurred during a clinical trial for CRSwNP,^9^ although the details are not available. Two cases of EGPA less likely to be associated with dupilumab (1 developing more than 300 days after a single dose of dupilumab and the other developing 5 months after discontinuation of dupilumab) were observed in patients with asthma or CRSwNP.^8^^,^^9^ In contrast, not a single case of EGPA has been reported in patients treated with dupilumab for eczema. These findings suggest that dupilumab alone does not cause EGPA but instead promotes the emergence of latent EGPA manifested as severe asthma or CRSwNP.

Dupilumab is a highly effective treatment for severe eosinophilic asthma and CRSwNP; however, these conditions can precede EGPA. Therefore, until biomarkers that can identify latent EGPA become available, clinical course should be carefully monitored after dupilumab is introduced, especially when systemic corticosteroids are tapered.
